# Supplementary material for: Alpha phase coding supports feature binding during working memory maintenance
Source: Commun Biol. 2026 May 4;9:922. doi: 10.1038/s42003-026-10071-9 (PMC13346530; doi:10.1038/s42003-026-10071-9)
Supplement: Supplementary file 1 — Supplementary Information [file 42003_2026_10071_MOESM1_ESM.pdf]

Supplementary Information

Alpha phase coding supports feature binding during working memory maintenance

Mattia F. Pagnotta[1,*], Aniol Santo-Angles[2], Ainsley Temudo[2,3], Joao Barbosa[4,5], Albert Compte[4], Mark D'Esposito[1,6], Kartik K. Sreenivasan[2,7]

[1] Helen Wills Neuroscience Institute, University of California Berkeley, Berkeley, CA, United States of America

[2] Division of Science and Mathematics, New York University Abu Dhabi, Abu Dhabi, United Arab Emirates

[3] University of Utah, Salt Lake City, UT, United States of America

[4] Institut d'Investigacions Biomèdiques August Pi i Sunyer (IDIBAPS), Barcelona, Spain

[5] Laboratoire de Neurosciences Cognitives et Computationnelles, INSERM U960, École Normale Supérieure - PSL Research University, 75005 Paris, France

[6] Department of Psychology, University of California Berkeley, Berkeley, CA, United States of America

[7] Center for Brain and Health, New York University Abu Dhabi, Abu Dhabi, United Arab Emirates

[*] Correspondence and material requests should be addressed to M.F.P. (email: pagnotta@berkeley.edu).

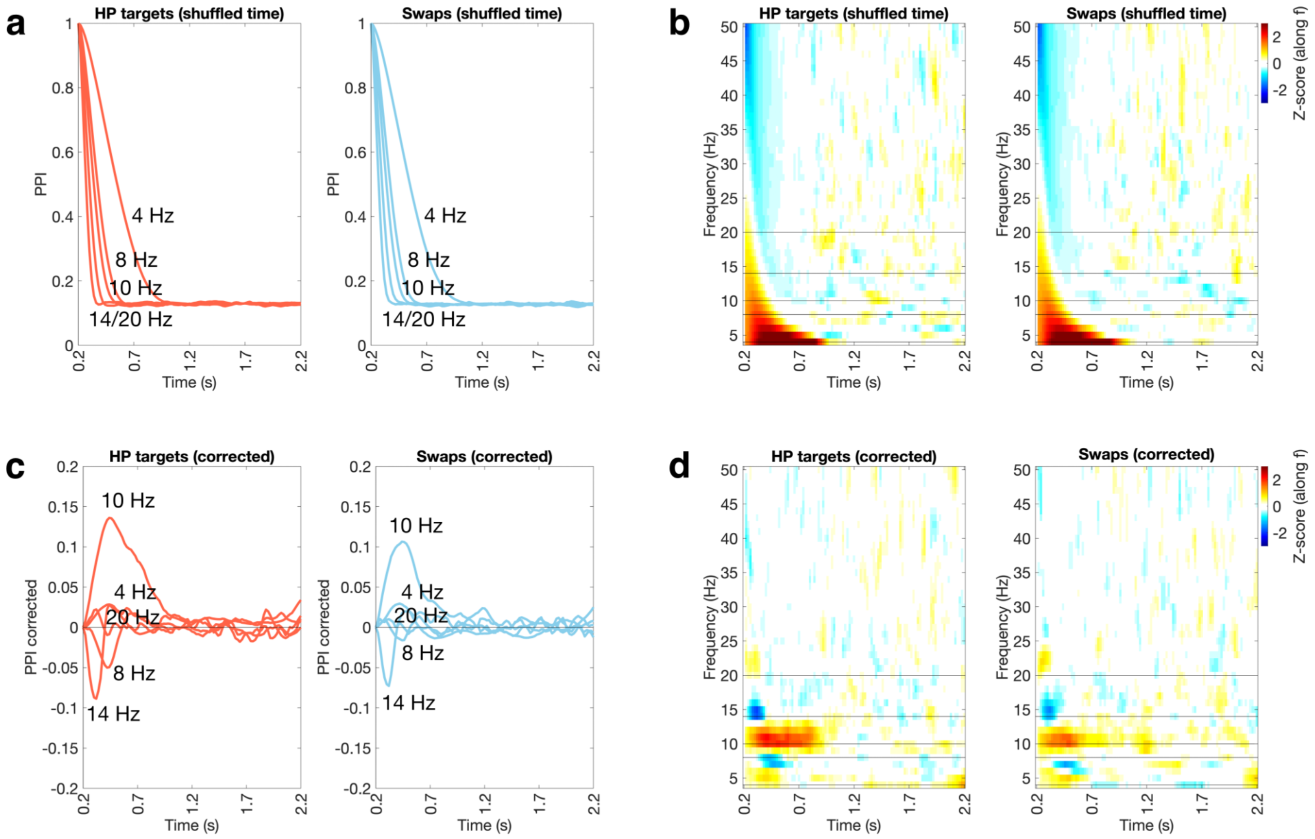

Fig. S1 | PPI estimates between HP targets and swaps. a Time course of PPI obtained from shuffling the signals over time in HP targets (left) and swaps (right) at different frequencies, highlighted by the black horizontal lines in the next panel. **b** PPI (shuffled signals over time): z-scores along frequencies for each time point in the delay, in HP targets (left) and swaps (right). **c** Time course of PPI corrected in HP targets (left) and swaps (right) at different frequencies, highlighted by the black horizontal lines in the next panel. **d** PPI corrected: z-scores along frequencies for each time point in the delay, in HP targets (left) and swaps (right).

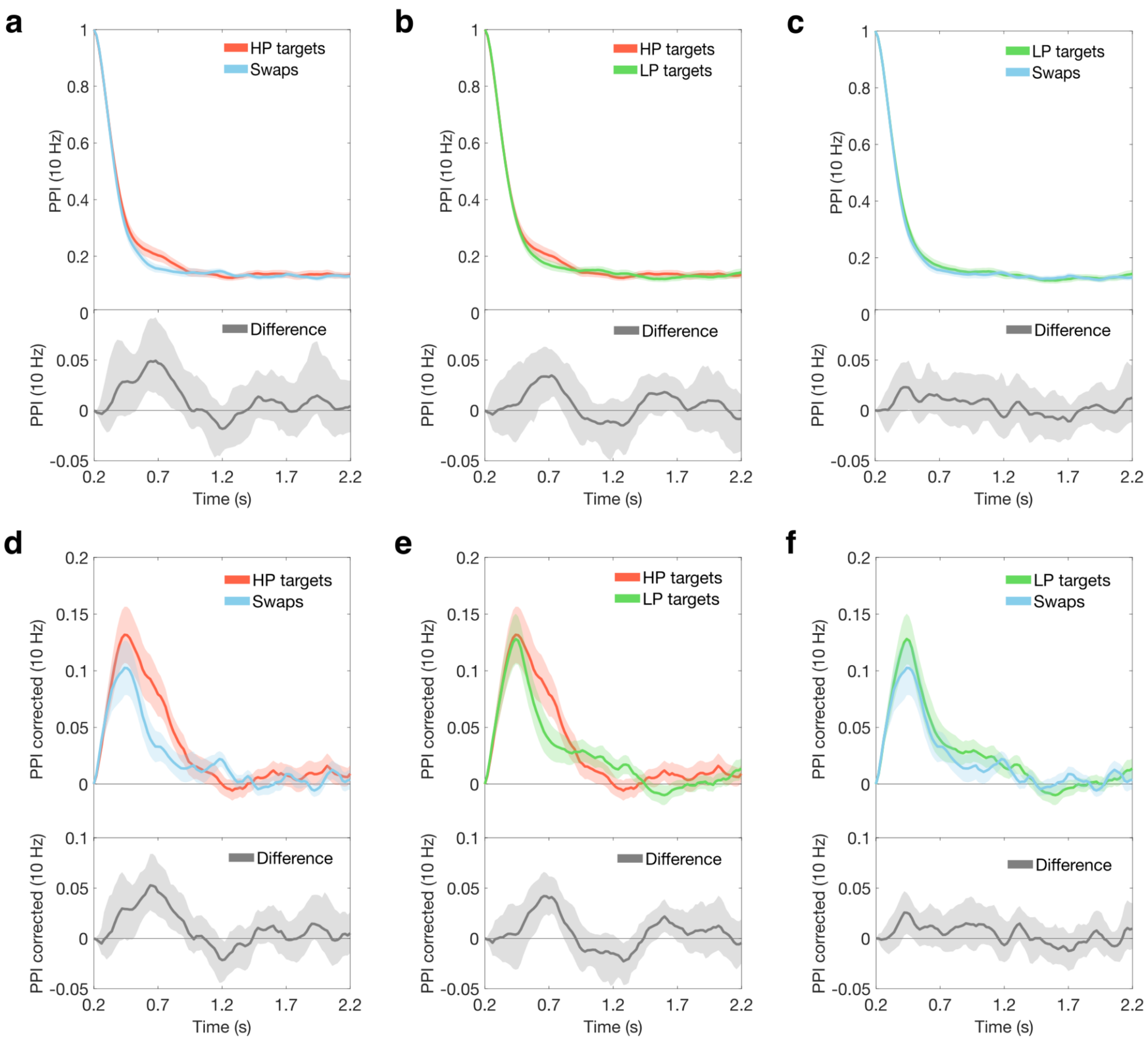

Fig. S2 | PPI estimates across MEG sensors of the cluster in the observed data, for the different trial types. **a** Time course of PPI at 10 Hz for HP targets (red) and swaps (blue), together with the difference between the two (gray). **b** Time course of PPI at 10 Hz for HP targets (red) and LP targets (green), together with the difference between the two (gray). **c** Time course of PPI at 10 Hz for LP targets (green) and swaps (blue), together with the difference between the two (gray). **d** Time course of PPI corrected at 10 Hz for HP targets (red) and swaps (blue), together with the difference between the two (gray). **e** Time course of PPI corrected at 10 Hz for HP targets (red) and LP targets (green), together with the difference between the two (gray). **f** Time course of PPI corrected at 10 Hz for LP targets (green) and swaps (blue), together with the difference between the two (gray). In each panel, the colored shadings (top) represent the standard error of the mean, while the gray shading (difference; bottom) represents 95% confidence intervals (CIs). CIs were estimated using the bias-corrected and accelerated method on a bootstrap distribution of across subjects differences, obtained by resampling with replacement 10,000 times.

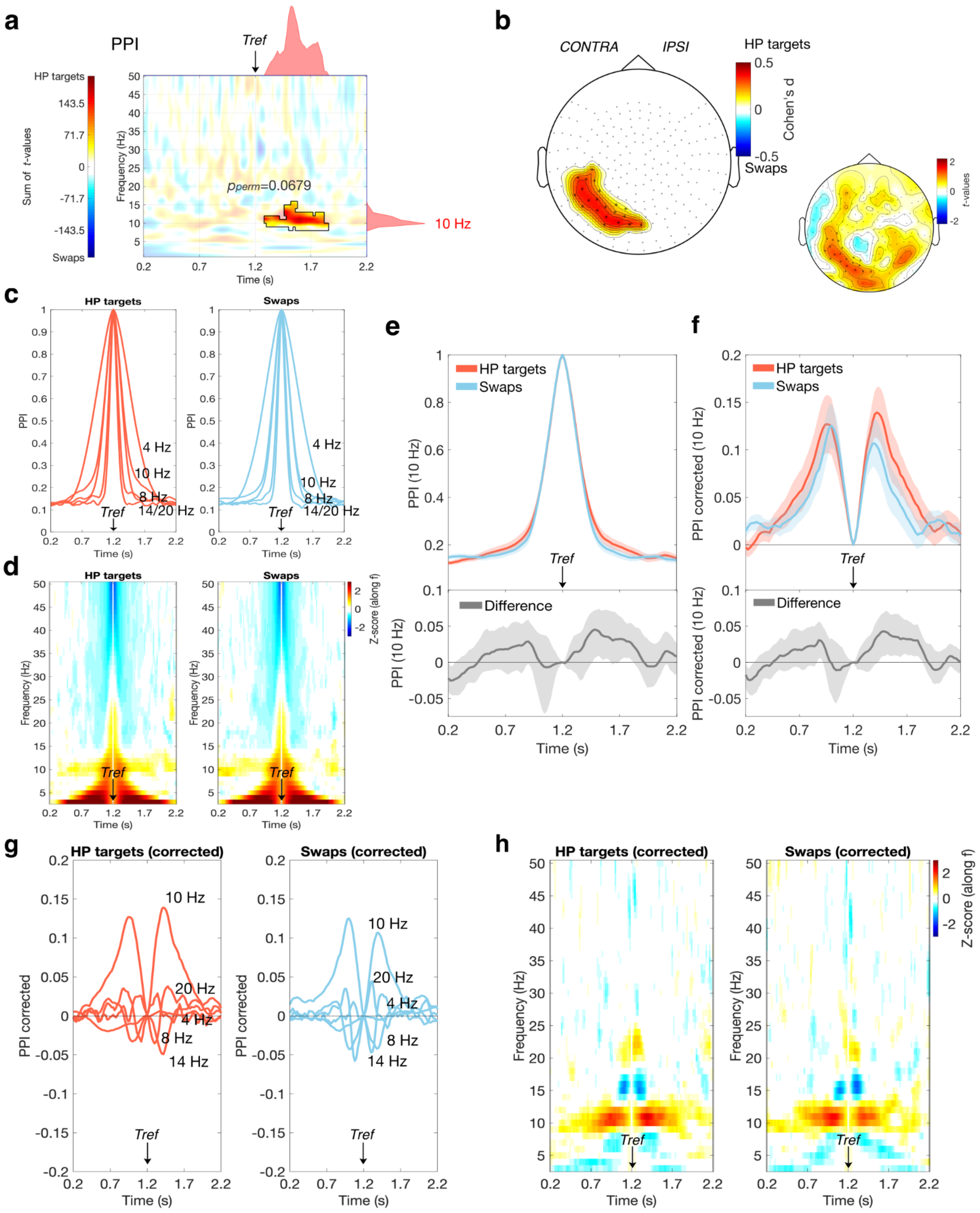
a
PPI
Tref
HP targets
Swaps
Sum of t-values
Frequency (Hz)
Time (s)
pperm=0.0679
10 Hz
b
CONTRA
IPSI
HP targets
Swaps
Cohen's d
t-values
c
HP targets
Swaps
PPI
4 Hz
10 Hz
8 Hz
14/20 Hz
d
Z-score (along f)
e
HP targets
Swaps
PPI (10 Hz)
Difference
f
PPI corrected (10 Hz)
g
HP targets (corrected)
Swaps (corrected)
PPI corrected
20 Hz
14 Hz
h
Frequency (Hz)
Time (s)

Fig. S3 | PPI differences between HP targets and swaps: analysis using the middle of the delay as the reference time point (t_{ref}=1.2 s) for PPI estimation. a Time-frequency distribution of the sum of PPI differences across MEG sensors (*t*-values). The black contour highlights the positive cluster found in the observed data. The marginal plots on the right and on top represent respectively the time-collapsed frequency distribution and frequency-collapsed time distribution of the differences between trial types. **b** Topography plot with superimposed effect sizes of PPI differences between HP targets and swaps, for each MEG sensor of the observed cluster (*t*-values are shown on the smaller topography plot on the right). The positive cluster did not reach statistical significance in the cluster-based permutations testing (p_{perm}=0.0679) **c** Time course of PPI in HP targets (left) and swaps (right) at different frequencies (as in Fig. 3). **d** Z-scores along frequencies for each time point in the delay, in HP targets (left) and swaps (right). **e** Time course of PPI estimates at 10 Hz for HP targets (red) and swaps (blue), together with the difference between the two (gray). **f** Time course of PPI corrected at 10 Hz for HP targets (red) and swaps (blue), together with the difference between the two (gray). In e–f, the red and blue shadings (top) represent the standard error of the mean, while the gray shading (difference; bottom) represents 95% confidence intervals (CIs). CIs were estimated using the bias-corrected and accelerated method on a bootstrap distribution of across subjects differences, obtained by resampling with replacement 10,000 times. **g–h** Same as in c–d, but for PPI corrected.

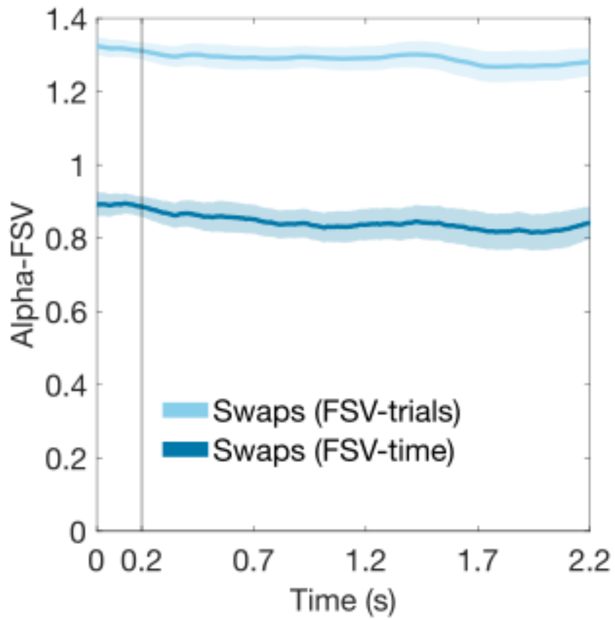

Fig. S4 | Measures of alpha-FSV in swaps. The time course of the average alpha-FSV across sensors of the cluster found in the data (see Fig. 5b) is shown for alpha-FSV over trials (light blue) and alpha-FSV over time (darker blue). The shadings represent the standard error of the mean.

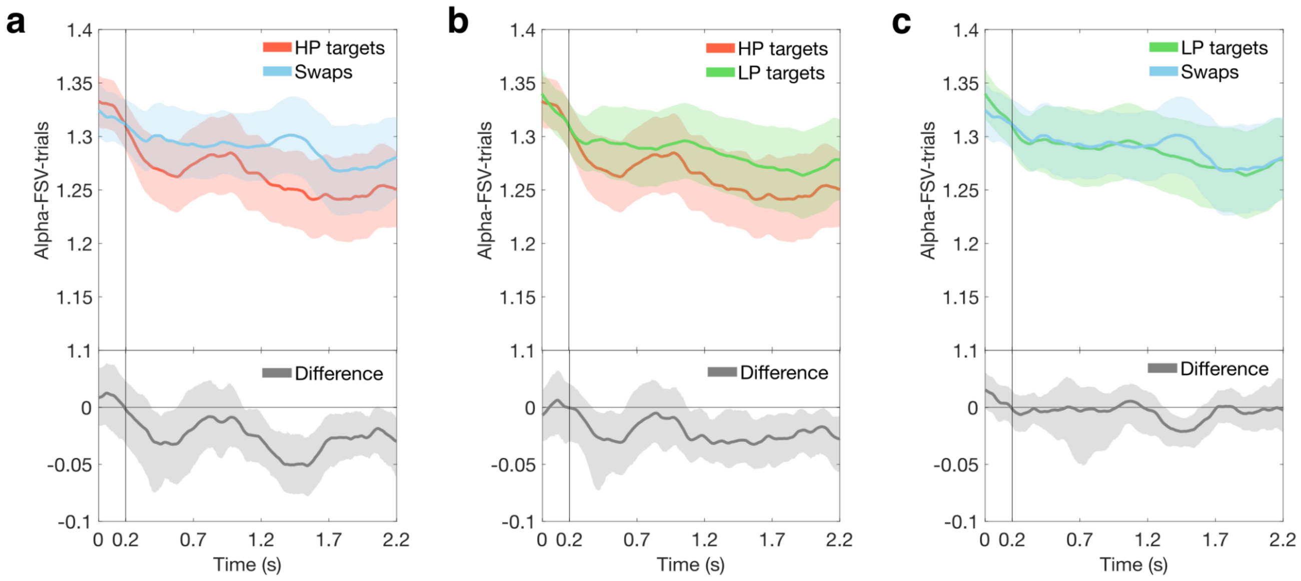

Fig. S5 | Alpha-FSV over trials across MEG sensors of the cluster in the observed data, for the different trial types. a Time course of alpha-FSV over trials for HP targets (red) and swaps (blue), together with the difference between the two (gray). **b** Time course of alpha-FSV over trials for HP targets (red) and LP targets (green), together with the difference between the two (gray). **c** Time course of alpha-FSV over trials for LP targets (green) and swaps (blue), together with the difference between the two (gray). In each panel, the colored shadings (top) represent the standard error of the mean, while the gray shading (difference; bottom) represents 95% confidence intervals (CIs). CIs were estimated using the bias-corrected and accelerated method on a bootstrap distribution of across subjects differences, obtained by resampling with replacement 10,000 times.
